# Bean Type Strongly Drives Specific Mechanical Energy and Distinguishes Extrudate Expansion During Extrusion Processing

**DOI:** 10.1111/1750-3841.71537

**Published:** 2026-10-09

**Authors:** Aniket Kamboj, Aimé Roger Raherison, Sahil Nain, Girish M. Ganjyal

**Affiliations:** ^1^ School of Food Science Washington State University Pullman Washington USA; ^2^ School of Bioresources and Technology King Mongkut University of Technology Thonburi Bangkok Thailand

**Keywords:** bean flour, color, expansion, multivariate analysis, specific mechanical energy, texture

## Abstract

This study examined how bean type and extrusion conditions influence process behavior and quality attributes of expanded extrudates produced from pre‐cooked pinto, navy, and black bean flours. Blends were extruded at three feed moisture levels (14%, 17%, and 20%, wet basis), and three screw speeds (100, 200, and 300 rpm). Product properties were strongly dependent on processing conditions and bean type. Navy beans produced more expanded and softer extrudates with expansion ratios of 1.58–2.49 and hardness values of 1.54–8.63 kg compared with pinto bean extrudates, which showed lower expansion (1.30–1.88) and higher hardness (2.62–15.55 kg). Black bean extrudates showed intermediate behavior, with expansion ratios of 1.68–2.11 and hardness values of 2.44–11.65 kg. Color change increased with more severe processing conditions for all bean types. Water solubility index (WSI) was strongly and positively correlated with specific mechanical energy (SME) for all bean flours, with Pearson correlation coefficients of r ≥ 0.97 for navy, pinto, and black beans. Scanning electron microscopy (SEM) images supported these findings, showing condition‐ and bean‐dependent cellular structures ranging from more uniform foams to coalesced pores and locally compact regions. K‐means clustering revealed three distinct extrusion product groups driven by bean type and processing intensity, distinguishing low‐energy, low‐expansion black and pinto bean extrudates from high‐viscosity and high‐SME navy bean formulations. Overall, the results show that bean type significantly affects structure development and energy demand during extrusion processing, requiring bean‐specific process optimization.

## 1 Introduction

Pulse‐based ingredients are increasingly being incorporated into extruded foods mainly because of their high protein value, fiber content, other nutrient density, and sustainable properties (Cotacallapa‐Sucapuca et al. 2021; Ek, Baner et al. 2020; Jeganathan and Ganjyal 2026). The three major dry bean market classes are pinto, black, and navy beans. In the United States, pinto, black, and navy beans remain major dry bean market classes, with 2025 production of 9.60, 7.15, and 3.14 million hundredweight, respectively (USDA National Agricultural Statistics Service, 2026). These beans can be milled and used as commercial ingredients in bakery, pasta, snacks, cereal, gluten‐free foods, and extruded products because they contribute protein, fiber, water‐holding capacity, viscosity, and structure (Thakur et al. 2019). Despite their increasing importance for gluten‐free, clean‐label, and high‐protein product formulation, whole pulse‐based formulations still receive relatively little attention in extrusion studies (Pietrysiak et al. 2020). The primary focus of these studies was on pulse flour and cereal starch mixtures (corn or rice starch) to enhance expansion properties, reduce melt viscosity, and improve bubble stabilization processes. For example, Dogan and co‐authors reported the development of an expanded extruded product using blends of lentil flour, corn starch, and corn oil, identifying a formulation dominated by lentil flour (67%), with corn starch (30%), and corn oil (3%) as the most suitable for expansion (Dogan et al. 2013). Our previous study suggests that incorporating mung bean protein into corn starch dramatically influences the puffing characteristics (Bernin et al. 2024). In brief, the addition of bean flour to starch in extrusion processes introduces proteins and other components that influence both the functional and structural properties of the extrudates. Therefore, the problem becomes more complex when whole bean flours are involved, due to their relatively higher fiber and protein contents, which interfere with starch melt rheology, reduce expansion, and impair structure formation compared to cereal systems with higher starch contents.

Bean flours usually have higher water absorption capacity, and the main factor that contributes to this is their high dietary fiber and protein content, as well as the strong entrapment of starch granules within their protein and fiber matrices. Studies on the water absorption capacity of navy bean, white bean, and broad bean flours showed that bean flours always absorb higher amounts of water compared to cereal flours, meaning they always have high values of water absorption index (WAI) (Ferawati et al. 2019; Patrascu et al. 2017). Thermal processes such as roasting and extrusion can weaken cellular structures and assist the solubilization of low‐molecular‐weight compounds, thereby improving hydration but reducing radial expansion (Stone et al. 2019, 2021). Improved water absorption results in limited expansion and a firmer texture in the final product (Stone et al. 2019). Thermal processing can also induce an observable color change, as they become less light and take on a brown hue. This is attributed to Maillard reactions (Ferawati et al. 2019; Stone et al. 2021).

The extrusion‐assisted puffing process is governed by the expansion of viscoelastic melt, followed by rapid solidification and stabilization of the expanded structure. Growth and stabilization of cavitation bubbles are completed at the die exit. All physical changes occurring during this process determine the characteristics of the final product. These conditions can be influenced by process parameters and the composition/structure of the raw materials used (Ding et al. 2005). Starch from cereals such as wheat, rice, maize, and potato has been well studied and utilized in food systems (Ganjyal et al. 2003; Kowalski et al. 2015). In contrast, for pulse and legume flours, starch is embedded within a complex matrix of protein, dietary fiber, and minerals. Whole legume flours exhibit a wide variation in pasting parameters such as water absorption, solubility, and functionality of starch and protein (Du et al. 2014). In addition, the dietary fiber in flours can have significant effects on the extrusion process and the characteristics of the final product, including volume shrinkage, higher density, and hardness due to the limited compatibility of insoluble fiber with the starch continuous phase (Robin et al. 2012). Variations have been reported for the starch‐related carbohydrates in different beans, ranging from 40% to 70%, with differences in resistant starch content, amylose content, and dietary fiber components, including cellulose, hemicellulose, and oligosaccharides (Siva et al. 2019). Previous studies demonstrated that pinto, navy, and black bean flour blends can be extruded into expanded puffs (Simons et al. 2015). Hardness, expansion, and sensory acceptability of bean puffs varied with bean type; however, limited discussion was provided on other contributing factors, including interactions between bean type and processing parameters and physicochemical properties. In particular, limited information is available on how bean flours respond to different feed moisture and screw speed conditions, and whether differences in extrusion process response, expansion, density, and texture can be interpreted through measured differences in proximate composition and thermal‐mechanical analysis.

More specifically, high‐fiber and high‐protein flour extrusion of pulses is significantly influenced by the interactions among their composition, water content, screw speed, and temperature, which govern torque, die pressure, and specific mechanical energy (SME) (Ek et al. 2021; Li et al. 2017). SME is considered an essential indicator of heat and mechanical input and is used to assess structure formation. Generally, it is considered that low SME results in low expansion, and high SME results in higher hardness and color loss (Ek, Kowalski et al. 2020). Water content has different behaviors depending on the threshold values, and lowers viscosity and SME due to plasticizing at low‐moderate water content, but inhibits structure formation at high water content (Kowalski et al. 2018). Since expansion, texture, color, and solubility index are influenced by interactions of two or more variables simultaneously, multivariate techniques can be applied for the interpretation of extrusion process characteristics of pulse products (Lee Rodgers and Nicewander 1988; Lloyd 1982). Therefore, this study evaluated the effects of bean type, feed moisture, and screw speed on SME and the physical properties of directly expanded bean flour extrudates. We hypothesized that bean type would significantly affect SME and extrudate quality due to inherent differences in flour composition and functionality, thereby leading to bean‐type‐dependent differences in expansion and texture.

## 2 Materials and Methods

### 2.1 Materials

Black bean, pinto bean, and navy bean flours used in this study were provided by Archer‐Daniels‐Midland (Decatur, IL, USA). All flours were manufactured pre‐cooked, as per the information provided by the manufacturer, although not completely pregelatinized. The use of precooked flours was selected to reflect commercially available bean flour ingredients used in expanded snack applications. Because precooking can alter flour functionality, the received flours were characterized before extrusion for proximate composition, thermal transition behavior, pasting behavior, WAI, and WSI. The moisture content of the pre‐cooked flours was measured using a convection oven according to the AACC International method 44‐15.02. Ash content was determined using AOAC Official Method 942.05. Fat content was determined based on AACC International Method 30‐25.01. The protein content was estimated using Rapid Max N exceed (Elementar Analysensysteme GmbH, Langenselbold, Germany) with an N_2_ conversion factor of 6.25. An ANKOM automated IDF/SDF analyzer (Model TDF, ANKOM Technology) was used to measure the IDF (insoluble dietary fiber) and SDF (soluble dietary fiber) proportions of each constituent using method AACC 32‐07.01. The total of IDF and SDF was used to compute TDF (total dietary fiber). This specific technique, often referred to as AOAC 991.43, was selected because it offers a distinct quantification of insoluble and soluble dietary fiber. Further details of the method can be found in our previous study (Wagner et al. 2024). All of the data for the three bean flours is given in Table 1. All flours had a particle size such that 95% passed through a #80 mesh sieve. All samples were properly sealed and stored at room temperature (25°C) until further extrusion processing.

**TABLE 1:** Proximate composition, thermal properties, color parameters, and functional indices of pinto, navy, and black bean flours.

Parameter	Pinto bean	Navy Bean	Black Bean
M.C (% w.b.)	7.36 ± 0.06^b^	7.10 ± 0.03^a^	7.08 ± 0.01^a^
Protein (%)	21.10 ± 0.11^b^	21.75 ± 0.05^a^	21.77 ± 0.04^a^
DCH (%)	40.24 ± 1.44	44.63 ± 3.34	41.7 ± 3.29
TDF (g/100 g)	25.65 ± 1.06^a^	22.55 ± 1.48^a^	25.25 ± 0.92^a^
IDF (g/100 g)	18.15 ± 0.35^a^	16.55 ± 0.49^b^	18.75 ± 0.07^a^
SDF (g/100 g)	7.55 ± 0.64^a^	6.00 ± 0.99^a^	6.50 ± 0.99^a^
Ash (%)	3.70 ± 0.97^a^	2.71 ± 0.25^a^	2.86 ± 0.01^a^
Fat (g/100 g)	1.95 ± 0.03^b^	1.26 ± 0.01^a^	1.34 ± 0.04^a^
T_o_ (°C)	144.46 ± 0.58^b^	142.39 ± 0.35^a^	141.77 ± 0.64^a^
T_p_ (°C)	144.95 ± 0.65^b^	142.87 ± 0.38^a^	142.17 ± 0.58^a^
∆H (J/g)	13.31 ± 1.17^a^	12.04 ± 2.87^a^	11.72 ± 3.14^a^
T_g_ (°C)	122.79 ± 2.06^a^	121.57 ± 0.99^a^	121.70 ± 1.05^a^
L^*^	85.16 ± 0.64^a^	90.24 ± 0.74^b^	76.26 ± 0.38^c^
a^*^	2.85 ± 0.09^a^	1.43 ± 0.09^b^	2.07 ± 0.02^c^
b^*^	10.56 ± 0.24^a^	16.51 ± 0.35^b^	8.36 ± 0.90^c^
WAI	3.13 ± 0.03^b^	3.58 ± 0.02^a^	3.18 ± 0.03^b^
WSI	12.13 ± 0.05^b^	10.62 ± 0.30^a^	12.07 ± 0.09^b^

Abbreviations: ΔH, enthalpy of transition; 100 – (%M.C + %Protein + %Fat + %Ash + %TDF); a^*^, red‐green color axis; b^*^, yellow‐blue color axis; DCH: digestible carbohydrates = L^*^, Lightness; T_g_, glass transition temperature; (T_o_), onset temperature of the DSC endothermic peak; (T_p_), peak temperature of the DSC endothermic peak; IDF, insoluble dietary fiber; M.C, (moisture content); SDF, soluble dietary fiber; TDF, total dietary fiber; WAI, water absorption index (g water/g dry solids); WSI, water solubility index (%).

Values are reported as mean ± standard deviation. Values followed by different superscript letters within the same row are significantly different (*P < 0.05*).

### 2.2 Moisture Adjustment and Feed Calibration

All formulations were conditioned to target moisture contents of 14% ± 0.5%, 17% ± 0.5%, or 20% ± 0.5% (wet basis). Please note that throughout the manuscript, moisture references are made on a wet basis, unless otherwise specified. Moisture adjustment was achieved by spraying deionized water continuously onto the flours while mixing in a dough mixed with a B flat beater type paddle mixer at the lowest speed setting of 1 (out of 3), which corresponds to less than 100 rpm (Model #A‐200, Hobart Manufacturing Co., Troy, OH, USA). Mixing was carried out for 5 min to ensure uniform water distribution. The hydrated blends were then sealed in polyethylene bags and stored at 4°C for 24 h to allow moisture equilibration throughout the material. The samples were removed from cold storage after equilibration and allowed to reach room temperature prior to extrusion processing. For extrusion, dry flour blends were fed into a hopper positioned at the inlet of barrel zone 1 (Figure 1) using a mass‐calibrated twin‐auger volumetric feeding system (Type 15‐37‐000, Brabender Instruments Inc., Duisburg, Germany). The volumetric feeding was calibrated separately for each formulation, and regression models were developed to relate flour flow rate (g/min) to feeder motor frequency (Hz).

**FIGURE 1:**
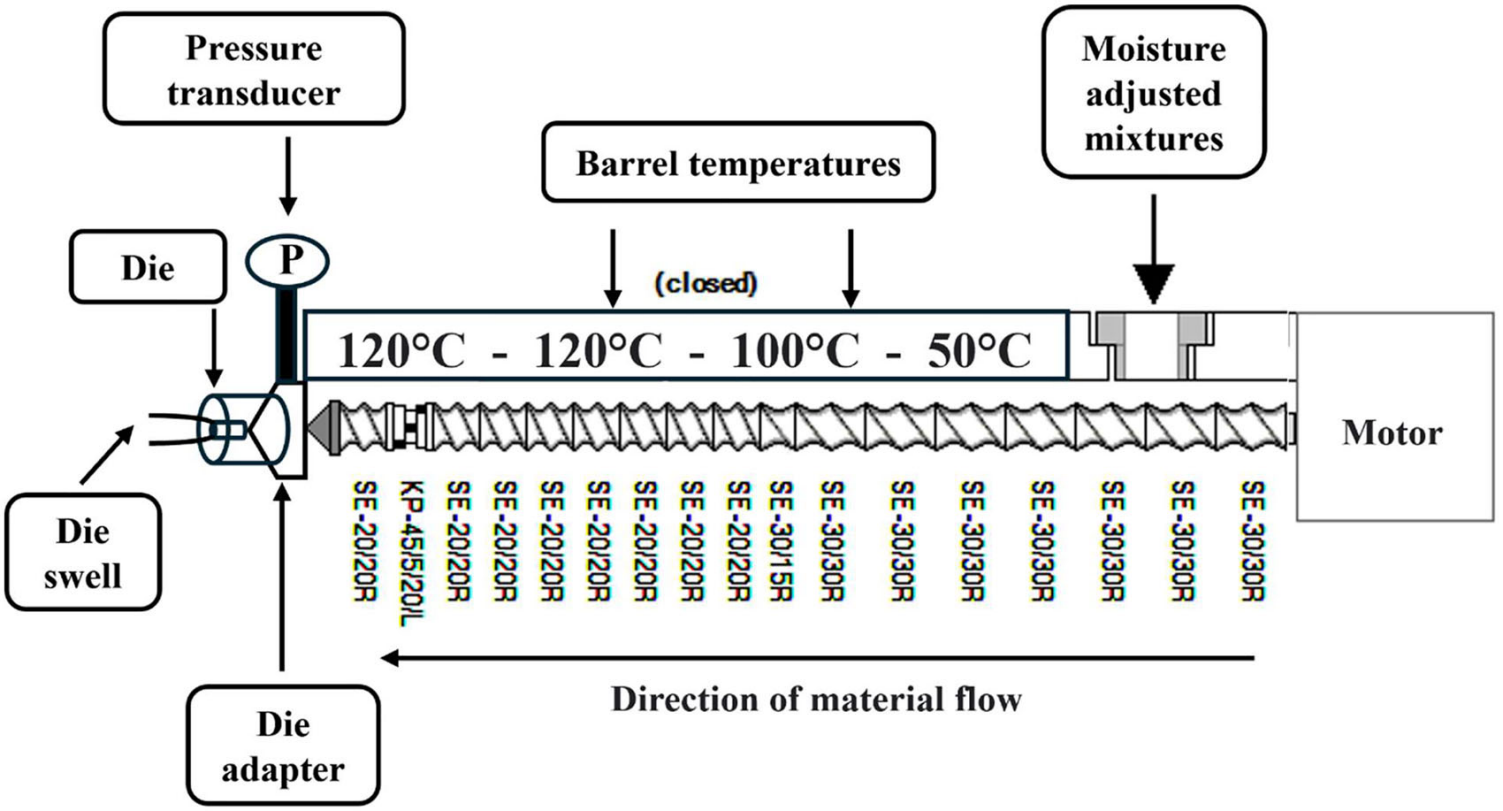
Twin‐screw extruder setup and screw profile used for low‐moisture (14%, 17%, and 20% w.b.) bean flour extrusion with a 3.16 mm die.

### 2.3 Thermal and Pasting Properties

The pre‐extrusion blends prepared in Section 2.2 were analyzed for thermal analysis using differential scanning calorimetry (DSC 25, TA Instruments Inc., New Castle, DE, USA). The instrument was operated with an airflow rate of 30 mL/min and a nitrogen purge rate of 319 mL/min. The system was calibrated with indium as the standard. About 3–4 mg of each sample was weighed and sealed in aluminum Tzero pans with hermetic lids (Tzero Hermetic Pan, TA Instruments Inc., New Castle, DE, USA). The DSC protocol followed the method described by Kamboj et al. (2025). The samples were scanned from 30 to 160°C during the first heating cycle at 10°C/min, using an empty sealed pan as the reference. Thereafter, the data analysis was done using Universal Analysis 2000 software, version 4.5A (TA Instruments Inc., New Castle, DE, USA). For all samples, the onset temperature (To), peak temperature (Tp), and enthalpy of transition (ΔH) were judged from peak analysis, and glass transition temperature (Tg) was reported at the inflection point during phase shift, as shown in Figure 2.

**FIGURE 2:**
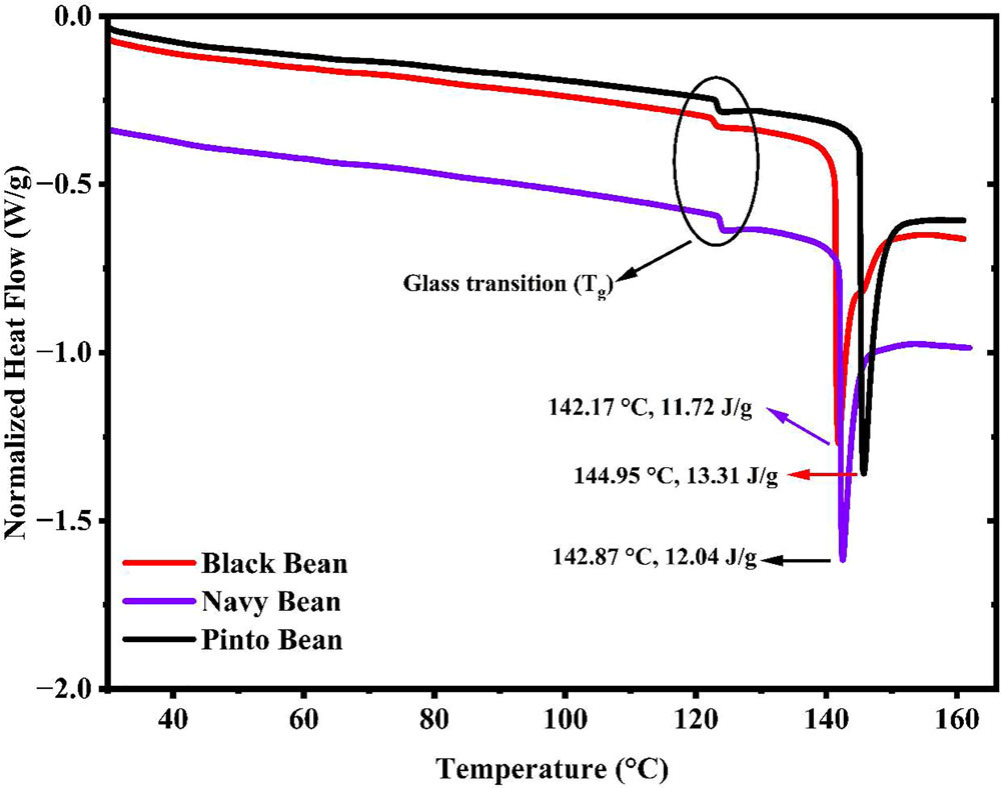
Differential scanning calorimetry (DSC) thermograms for black, navy, and pinto bean flours at 20% moisture.

Pasting analysis was done using the Viscoamylograph Machine or ViscoQuick (Brabender GmbH & Co., Dulsburg, Germany) at 83.2% moisture content (w.b.) using the following temperature profile: samples were equilibrated at 25°C for 1 min, heated to 93°C at 10°C/min, held at 93°C for 5 min, cooled to 50°C at 10°C/min, and then held at 50°C for 1 min. At all stages, the hydrated samples were blended at 160 rpm (Ek et al. 2021).

### 2.4 Experimental Design and Process Parameters

The extrusion trials were conducted using a co‐rotating twin‐screw extruder (Model TSE 20/40, 7.5 HP, Brabender GmbH & Co. KG, Duisburg, Germany) with a 20:1 length‐to‐diameter ratio and a screw diameter of 20 mm (Kowalski et al. 2018). The detailed screw profile and twin‐screw extruder setup are shown in Figure 1. The figure shows the twin‐screw extruder configuration and the screw profile used for pulse flour extrusion under low‐moisture conditions (14‐20%). The attached die has an orifice diameter of 3.16 mm for expansion/puffing applications. The screw elements consist of different types, including SE‐30/30R, SE‐20/20R, and KP‐45/5/20L. The nomenclature system uses SE to identify standard screw elements and KP to identify kneading paddles; the first number in each pair indicates the diameter and pitch, and R/L indicates the screw direction.

The extrusion experiments were conducted using a full factorial design with three factors: bean type, feed moisture content, and screw speed. There were three bean types (black bean, navy bean, and pinto beans), three moisture contents (14%, 17%, or 20%, wet basis), and three screw speeds (100, 200, and 300 rpm). These processing conditions were based on the extrudability of these specific materials under finalized conditions, as revealed by multiple pre‐trials. The feed rate was fixed at 60 g/min for all trials. The experimental design produced 3 × 3 × 3 = 27 experiments using 100% bean flours. Each treatment combination was extruded in duplicate, giving a total of 54 extrusion runs. Torque, back pressure, and SME responses were calculated from the average of 30 data points (15 from each trial). The specific mechanical energy was calculated using equation (1) following Kamboj et al. (2025).

(1) $$\mathrm{SME}\;=\frac{N}{N_{max}}\;\cdot \frac{\tau }{\tau_{max}}\cdot \frac{P_{max}}{\dot{m}}$$

where SME is the specific mechanical energy (kJ kg^−1^), N is the screw speed (rpm), τ is the motor torque (Nm), ṁ is the mass feed rate (Kg s^−1^), P_max_ (5.59 kW) is the maximum motor power, N_max_ (1000 rpm), and τ_max_ (50 Nm) are extruder system limits.

### 2.5 Product Characterization

#### 2.5.1 Expansion Ratio

The radial expansion ratio (ER) was computed using the method previously reported by Wagner et al. (2023). A digital vernier caliper (Model DCLA‐0605, Clockwise Tools Inc., Valencia, CA, USA) was used to measure the extrudate diameter. The ER was computed by dividing the die orifice diameter by the average extrudate diameter. Across two extrusion trials, 25 cm strips were collected from each sample, and 8 strips were randomly selected for ER measurement. Each strip was examined at 8 randomly selected points, resulting in a total of 64 individual expansion measurements per sample.

#### 2.5.2 Hardness

The hardness of the expanded extrudates was evaluated using a Texture Analyzer (Model TA‐XT2i, Stable Micro Systems, Surrey, UK), following the methodological framework outlined in previous research (Boakye et al. 2022) with a few modifications. Before testing, the extrudates were cut into uniform cylindrical segments, each measuring 13 mm in length. The diameter varied based on the expansion ratio observed for each sample. Hardness testing involved a double‐compression cycle with the following parameters: pre‐test speed of 1.00 mm/s, test speed of 1.00 mm/s, and post‐test speed of 5.00 mm/s. The test was conducted in strain mode at 50% deformation, with a 5‐second interval between the two compressions. The force‐time curve generated during the test was used to determine the maximum force (F_max_) from the first compression, called the hardness value in this study. Each extrusion condition was tested in ten replicates (n = 20 in total for duplicate trials), and mean values were calculated for analysis.

#### 2.5.3 Color Analysis

A colorimeter (CM‐5, Konica Minolta) was used to measure the CIELAB criterion in accordance with the method by Kamboj et al. (2025). A zero‐calibration box (CM‐A124) and a white calibration plate (CM‐A210) were used to calibrate the device. The sample was measured in the L^*^, a^*^, and b^*^ color space using a 3 mm target mask. Four (n = 4) readings were taken for each sample. The resulting L^*^ (lightness), a^*^ (red‐green), and b^*^ (yellow‐blue) values were recorded. To measure the overall color difference between the treated samples and their corresponding controls, the ΔE values were computed. Equation (2) was used to calculate the ΔE values for each bean sample, using the corresponding raw unextruded flours as the reference.

(2) $$\Delta E=\sqrt{{\left(L^{*}-L_{0}^{*}\right)}^{2}+{\left(a^{*}-a_{0}^{*}\right)}^{2}+{\left(b^{*}-b_{0}^{*}\right)}^{2}}\;$$

where ​ $L_{0}^{*}$, $a_{0}^{*}$, and $b_{0}^{*}$, are the corresponding color parameters of the reference (unextruded bean flours).

### 2.6 Scanning Electron Microscopy (SEM)

The microstructural analysis of extrudates was performed using a Quanta 200F Field Emission Scanning Electron Microscope (FEI, Hillsboro, OR, USA) at the Franceschi Microscopy and Imaging Center (FMIC), Washington State University. Extrudates produced from pinto bean, navy bean, and black bean flours were dried in a convection oven at 45°C overnight to achieve a final moisture content of approximately 4% (w.b.). Dried samples were then cleaved using a sharp blade and mounted on aluminum SEM stubs using double‐sided carbon adhesive tape. The mounted samples were coated using a sputter coater (208HRD, Cressington Scientific Instruments, Watford, UK) under vacuum for 60 seconds to ensure conductivity. Micrographs were captured at magnifications of 25× and 50×. Representative images were used to assess internal pore structure and surface morphology.

### 2.7 Water Absorption Index (WAI) and Water Solubility Index (WSI)

Solubility behavior was evaluated using the WAI and water solubility index (WSI), following the method of Anderson et al. (1970). The extrudates were dried and milled into a fine flour. A 2.5 g sample was placed in a 50 mL centrifuge tube, and 30 mL of Milli‐Q water was added, then vortex‐mixed for 2 min to ensure uniform dispersion. The tubes were incubated in a water bath at 30°C for 30 min with occasional shaking. After incubation, the samples were centrifuged at 3000 × g for 10 min. The resulting supernatant was dried separately in aluminum pans at 105°C overnight. The weight of the leftover pellet was used to calculate WAI, while WSI was measured from the dried solids in the supernatant. The analysis was done in duplicate for each sample from two independent extrusion trials, yielding a total of 4 measurements per treatment (n = 4).

### 2.8 Univariate and Multivariate Statistical Analysis

All statistical analyses were carried out using OriginPro 2025b (OriginLab Corporation, Northampton, MA, USA). The mean and standard deviation were calculated for each process and product quality variable, based on 54 extrusion runs. For all univariate comparisons, statistical significance was established at *p* < 0.05. Before subjecting the data to multivariate analysis, checks were performed to assess reproducibility across replicates. Subsequently, variable scaling was performed to assign equal weights via z‐scoring (Table 3). A Pearson correlation matrix was generated in OriginPro to assess linear relationships between processing parameters (torque, back pressure, and SME) and product properties (expansion ratio, hardness, color components, WAI, and WSI). The correlation matrix was illustrated using a correlation heat map to identify dominant relationships between processes and properties, with a focus on those linked to SME. Unsupervised clustering analysis was carried out using K‐means clustering (McQueen 1967). The cluster analysis was done with three clusters (k = 3). The k‐means clustering algorithm was set to random seeding with auto initialization, a maximum iteration of 300, and a tolerance limit of 1 × 10^−4^ using the Standard Euclidean distance to assign points to clusters. For a data point 𝑥_𝑖_ = (𝑥_𝑖1_, 𝑥_𝑖2_,…,) and a cluster centroid μ_j_ = (μ_j1_, μ_j2_,…,μ_jp_), cluster assignment was based on minimizing Euclidean distance, computed as Equation (3).

(3) $$d^{2}\;\left(x_{i},\mu_{j}\right)=\sum_{k=1}^{p}{\left(x_{ik}-\;\mu_{jk}\right)}^{2}\;$$

Centroids were updated at each iteration as the arithmetic mean of all points in the cluster as per Equation (4).

(4) $$\mu_{j}=\frac{1}{n_{j}}\;\sum_{i\in C_{j}}x_{i}$$

The final results obtained from the cluster analysis included cluster coordinates (center coordinates), within‐cluster sum of squares, mean and max distances, and distances between cluster centroids (Supplementary file: Table S1, S2). The first two components were graphed together with clustering to show differences among bean types and processing parameters.

## 3 Results and Discussion

### 3.1 Composition and Physicochemical Differences Among Bean Flours

Table 1 showed the significant differences in extrusion relevant parameters of bean flours. Pinto bean showed the highest TDF, SDF, ash, fat, and thermal transition behavior, while navy bean showed the lowest dietary fiber values but the highest WAI. Compared with other flours, navy bean flour exhibited the highest WAI, indicating greater water‐absorption capacity but a lower soluble fraction, which may include SDF (see Table 1). The WAI and WSI values of flours are critical parameters to predict extrusion behavior (Anderson et al. 1970). The interaction of flour and water, swelling and solubilization, affect starch/protein plasticization and melt viscosity development during extrusion (Ganjyal 2020). The functional properties of whole legume flours reported in the literature in terms of WAI, WSI, pasting properties, and other characteristics for pinto, black, navy, and other legume flours have significant differences, which correlate with the differences in protein/starch ratio and other characteristics of these flours (Du et al. 2014). The molecular composition of starch (amylose/amylopectin ratio) was not investigated in this work. However, previous studies have reported that black, navy, and pinto beans contain comparable amounts of soluble and resistant starch, as well as amylose content (approximately 18%–20% g/100 g wet basis) (Hoover et al. 2010; Siva et al. 2019). Pinto bean starch has been reported to contain higher amylose than black and navy bean starches, which may contribute to greater melt strength and thermal stability during extrusion (Hoover et al. 2010; Hoover and Ratnayake 2002). However, the hardness and expansion results in the present study suggest that amylose content was not the only controlling factor. Differences in fiber content, hydration and solubilization behavior (WAI/WSI), and thermal properties likely played a larger combined role in determining extrudate structure and texture (Anderson et al. 1970; Cotacallapa‐Sucapuca et al., 2021; Ganjyal 2020).

### 3.2 Thermal and Pasting Properties Analysis

DSC analysis revealed distinct differences in thermal transition properties among black, navy, and pinto bean flours at a moisture level of 20% (Figure 2). In particular, all bean flours showed a shallow and broadly described endothermic transition occurring approximately around 120°C–130°C, which can be assigned to the glass transition (T_g_) of amorphous components of pulse flours (Kyomugasho et al. 2021). Legume flours usually contain complex matrices of proteins, amylose/amylopectin complexes, non‐starch polysaccharides, and lipids; hence, their T_g_ transitions can only be described vaguely and not well resolved, especially at intermediate moisture contents (Ratnayake and Jackson 2008; Slade and Levine 1995). Generally, these subtle T_g_‐type transitions were previously reported among other legumes like lentils, chickpeas, and beans when moisture was held at more than 15%, because at such moisture contents, the amorphous phase becomes plasticized and accompanies T_g_ shifts into the 100°C to 140°C range (Lefèvre et al. 2021, Lefèvre et al. 2022). This glass temperature range corresponds to the onset of matrix softening and starch‐protein interface mobilization, which can affect further extrusion behavior. These small differences in peak temperature and enthalpy values (13.31 J/g, 12.04 J/g, and 11.72 J/g for pinto, navy, and black bean, respectively) among the bean varieties are well within the range typically found for bean flours and have been attributed to minor variations in the crystallization characteristics of the starch and protein‐starch interactions, rather than to any specific structural differences (Chung et al. 2008; Hoover and Ratnayake 2002). In any case, the overlap of the thermograms among the different bean varieties clearly suggests that in similar moisture and thermal environments, the thermal transition properties of the examined bean flours are similar to each other and to previously reported values, exhibiting little cultivar dependence in DSC gelatinization characteristics among dry legume flours (Hoover et al. 2010). Overall, the thermal softening and gelatinization of the pinto, navy, and black beans during the uniform heating, as shown in the DSC results, were quite comparable to one another and thus can be classified into the same thermal transition behavior and can be further used for thermo‐mechanical processing in similar heating conditions.

The bean flours exhibited small viscosity changes at lower temperatures (Figure 3) before their viscosity levels increased significantly during the final cooling process. The black bean paste showed the highest viscosity during the final cooling part, while, thereafter, the navy bean and pinto bean followed in sequence as shown in Figure 3. The results showed that beans affect paste thickening and retrogradation patterns because they behaved differently when subjected to the same amount of moisture and shear conditions. Differences in pasting magnitude and setback among common beans are consistent with reported variation in legume starch characteristics and the influence of non‐starch components (protein/fiber) on swelling and paste development (Ek et al. 2021). Black bean flour showed the highest maximum viscosity despite its relatively high IDF and protein contents (Table 1). Its higher viscosity was more likely related to greater residual starch swelling capacity and paste‐building capacity in the hydrated flour matrix (Hoover and Ratnayake 2002). In contrast, the lower maximum viscosity of pinto bean flour may be related to its lower WAI, higher fat content, and starch showing a lower swelling factor and higher relative crystallinity for pinto bean starch (Table 1). These factors suggest more restricted swelling and greater resistance to paste development under the applied heating profile.

**FIGURE 3:**
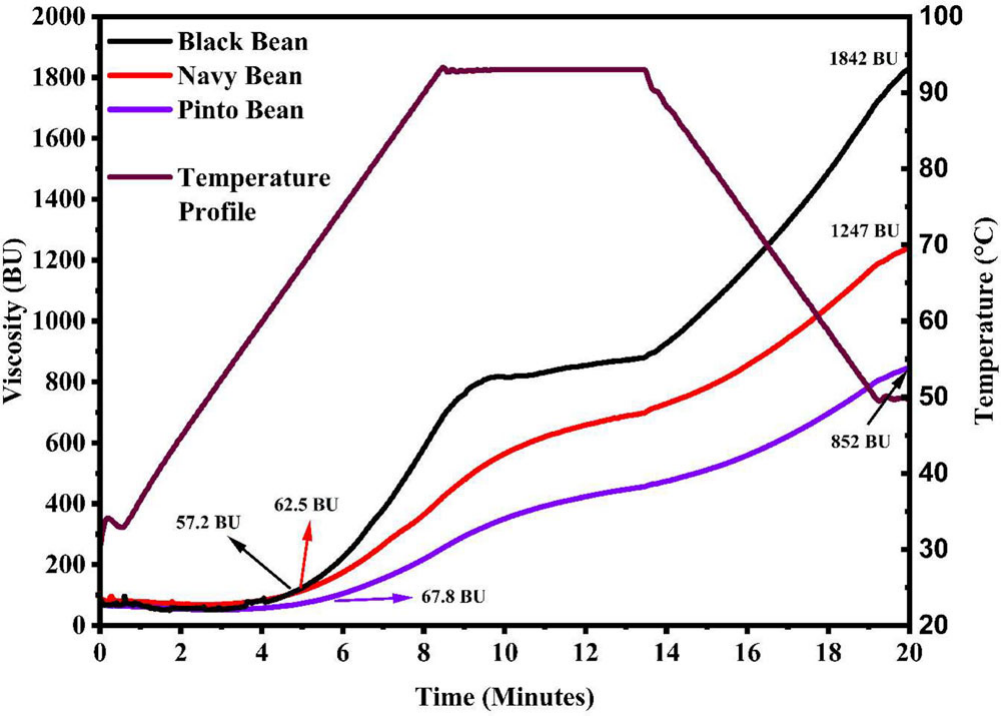
Pasting profiles of black, navy, and pinto bean flours measured using a Brabender Visco‐amylograph at 83.5% moisture content (wet basis).

### 3.3 Extrusion Processing Behavior

Table 2 clearly shows that torque reduced with the increase in moisture content and screw speed for all three types of bean flours. The value of the torque varied from 11.51 Nm to 42.60 Nm, the maximum value of the torque was found for the navy bean at 14% moisture content and at 100 rpm, and the minimum value was obtained for the pinto bean at 20% moisture content and 300 rpm. At a constant screw speed, increasing the moisture content from 14% to 20% decreased torque. A similar pattern was observed for black bean at 100 rpm: at 14% moisture content, the torque was 38.19 Nm, whereas at 20% moisture content, it was 23.08 Nm. At constant moisture content, increasing the screw speed from 100 rpm to 300 rpm resulted in a lower torque value. With the moisture percentage increasing from 14% to 20% under constant conditions, the torque value was found to decrease significantly. This is because water has a plasticizing effect on the mixture, resulting in the reduction of melt viscosity, thus reducing the resistance to the screw (Kamboj et al. 2025). A similar pattern was observed for the increased screw speed from 100 rpm to 300 rpm at constant moisture content; a reduction in the value of the torque was found. Generally, at constant moisture content, the increased screw speed can lead to increased viscous dissipation effects, resulting in the reduction of the apparent viscosity, thus reducing the resistance (i.e., the value of the torque), due to the increased shear rate (Fel et al. 2014a).

**TABLE 2:** Extrusion processing conditions, color attributes, functional indices, and cluster classification of bean extrudates.

Bean Type	M.C (%)	SS (rpm)	Torque (Nm)	WAI	WSI	L*	a*	b*	∆E	Cluster Group
Navy bean	14%	100	42.60 ± 0.97^a^	3.79 ± 0.10^cd^	19.69 ± 1.29^d^	80.31 ± 0.97^cd^	6.12 ± 0.72^bc^	22.84 ± 1.93^abc^	12.72 ± 1.97^cde^	I
		200	27.75 ± 0.96^c^	3.66 ± 0.07^de^	25.76 ± 1.00^b^	77.02 ± 0.71^ef^	7.77 ± 0.22^a^	25.56 ± 0.53^ab^	17.24 ± 0.58^ab^	III
		300	21.27 ± 0.24^e^	3.56 ± 0.08^e^	29.01 ± 0.68^a^	76.15 ± 0.97^f^	8.17 ± 0.29^a^	26.42 ± 0.83^a^	18.53 ± 0.40^a^	III
	17%	100	31.67 ± 1.07^b^	4.05 ± 0.15^b^	17.25 ± 0.38^e^	82.04 ± 0.53^bc^	5.24 ± 0.26^cd^	24.31 ± 1.37^abc^	11.97 ± 1.16^cdef^	I
		200	21.89 ± 1.22^e^	3.97 ± 0.13^bc^	20.25 ± 0.95^d^	80.91 ± 1.78^cd^	5.87 ± 0.93^bc^	25.12 ± 1.54^abc^	13.46 ± 2.50^bcd^	I
		300	17.59 ± 0.29^f^	3.76 ± 0.10^de^	22.94 ± 1.25^c^	78.64 ± 1.12^de^	6.99 ± 0.56^ab^	25.58 ± 0.78^ab^	15.74 ± 1.47^abc^	III
	20%	100	24.04 ± 0.55^d^	4.11 ± 0.12^ab^	14.83 ± 0.61^f^	84.54 ± 0.62^a^	4.00 ± 0.27^d^	21.81 ± 1.60^c^	08.25 ± 1.36^f^	I
		200	16.76 ± 0.50^f^	4.29 ± 0.09^a^	16.58 ± 0.45^d^	83.42 ± 1.06^ab^	4.42 ± 0.55^d^	22.05 ± 2.41^bc^	09.33 ± 2.43^ef^	I
		300	15.53 ± 1.56^g^	4.16 ± 0.08^ab^	19.46 ± 0.29^e^	82.53 ± 0.99^abc^	4.97 ± 0.49^cd^	22.29 ± 1.60^bc^	10.29 ± 1.81^def^	I
Pinto bean	14%	100	31.67 ± 1.06^a^	4.07 ± 0.04^a^	15.97 ± 0.26^c^	77.99 ± 0.26^a^	6.39 ± 0.16^cde^	16.09 ± 0.27^cd^	09.73 ± 0.28^cde^	II
		200	20.97 ± 1.78^c^	4.08 ± 0.03^a^	18.59 ± 1.10^b^	77.31 ± 1.73^ab^	6.88 ± 0.66^abcd^	17.25 ± 0.82^ab^	11.10 ± 1.93^bcd^	II
		300	19.49 ± 3.82^d^	4.08 ± 0.04^a^	21.60 ± 1.12^a^	74.69 ± 0.77^c^	7.66 ± 0.33^a^	18.10 ± 0.58^a^	13.78 ± 0.86^a^	III
	17%	100	26.36 ± 2.30^b^	4.05 ± 0.07^a^	15.40 ± 0.56^cd^	78.29 ± 0.47^a^	6.14 ± 0.24^de^	15.47 ± 0.31^de^	09.07 ± 0.61d^e^	II
		200	16.44 ± 1.53^e^	4.06 ± 0.04^a^	16.54 ± 0.86^c^	76.68 ± 0.38^abc^	6.74 ± 0.09^bcd^	16.17 ± 0.29^cd^	10.90 ± 0.23^bcde^	II
		300	15.52 ± 2.14^ef^	4.06 ± 0.04^a^	18.96 ± 0.91^b^	74.74 ± 1.53^c^	7.37 ± 0.37^ab^	16.98 ± 0.16^bc^	13.07 ± 1.3^ab^	II
	20%	100	20.10 ± 1.25^cd^	3.98 ± 0.04^a^	14.08 ± 0.29^d^	78.54 ± 0.99^a^	5.93 ± 0.25^e^	14.83 ± 0.45^e^	08.47 ± 0.95^e^	II
		200	14.72 ± 0.58^f^	4.07 ± 0.17^a^	15.09 ± 0.92^cd^	76.70 ± 1.13^abc^	6.53 ± 0.18^cde^	15.48 ± 0.14^cde^	10.48 ± 0.99^cde^	II
		300	11.51 ± 0.25^g^	4.11 ± 0.10^a^	15.93 ± 0.88^c^	75.71 ± 0.82^bc^	6.91 ± 0.41^abc^	16.02 ± 0.44^abc^	11.70 ± 1.10^abc^	II
Black bean	14%	100	38.19 ± 1.20^a^	4.06 ± 0.04^c^	19.95 ± 0.14^e^	69.31 ± 1.28^a^	5.96 ± 0.29^ab^	13.65 ± 0.22^b^	09.58 ± 1.15^ab^	II
		200	22.65 ± 0.51^c^	4.01 ± 0.07^cd^	23.37 ± 0.28^c^	69.81 ± 1.73^a^	6.28 ± 0.35^ab^	14.65 ± 0.38^ab^	10.00 ± 1.35^ab^	II
		300	19.85 ± 0.33^d^	3.68 ± 0.02^e^	27.59 ± 0.44^a^	70.61 ± 1.54^a^	6.65 ± 0.32^a^	16.16 ± 0.40^a^	10.70 ± 1.25^a^	III
	17%	100	29.30 ± 1.20^b^	4.06 ± 0.07^c^	17.59 ± 0.32^f^	70.91 ± 1.49^a^	4.62 ± 0.31^cd^	10.63 ± 0.71^cd^	06.39 ± 1.45^bc^	II
		200	18.71 ± 0.55^e^	4.01 ± 0.01^cd^	20.87 ± 0.17^d^	70.02 ± 1.85^a^	5.33 ± 0.11^bc^	11.79 ± 0.15^bc^	07.89 ± 1.53^abc^	II
		300	16.42 ± 0.68^f^	3.88 ± 0.02^d^	24.68 ± 0.19^b^	70.42 ± 1.13^a^	6.20 ± 0.41^ab^	14.00 ± 0.73^ab^	09.12 ± 1.35^ab^	III
	20%	100	23.08 ± 0.43^c^	4.25 ± 0.17^ab^	14.90 ± 0.29^g^	72.56 ± 1.59^a^	3.93 ± 0.36^d^	09.61 ± 0.84^d^	04.35 ± 1.77^c^	II
		200	16.26 ± 0.34^f^	4.34 ± 0.13^a^	17.57 ± 0.78^f^	70.28 ± 2.88^a^	4.87 ± 0.68^cd^	10.79 ± 0.86^cd^	07.07 ± 3.01^abc^	II
		300	13.52 ± 0.36^g^	4.13 ± 0.09^bc^	20.36 ± 0.40^de^	69.64 ± 2.23^a^	5.38 ± 0.55^bc^	11.78 ± 1.00^bc^	08.17 ± 2.41^abc^	II

Abbreviations: ΔE, total color difference; L*, a*, b*, CIELAB color coordinates; MC, moisture content (% w.b.); SS, screw speed; WAI, water absorption index; WSI, water solubility index.

Values are mean ± standard deviation. Different letters within a column indicate significant differences (Tukey's HSD, p < 0.05). Cluster groups were determined by k‐means clustering.

Additionally, SME rose with screw speed and decreased with feed moisture for all three types of bean flours, from 224.69 to 713.38 kJ/kg [Figure 4a,b,c]. Increasing the screw speed from 100 rpm to 300 rpm under constant moisture conditions further increased SME for each bean type. For each screw speed, increasing moisture from 14% to 20% lowered SME, indicating that, with increasing plasticity, the mechanical energy input per unit mass decreases. The value of SME for all four types of bean flours rose with increasing screw speed and decreased with increasing moisture content, in accordance with what has been previously observed in extruder systems, wherein increasing input of mechanical energy per unit mass is promoted by screw speed, but water‐induced plasticization opposes this process (Singha et al. 2018; Yeh and Jaw 1999).

**FIGURE 4:**
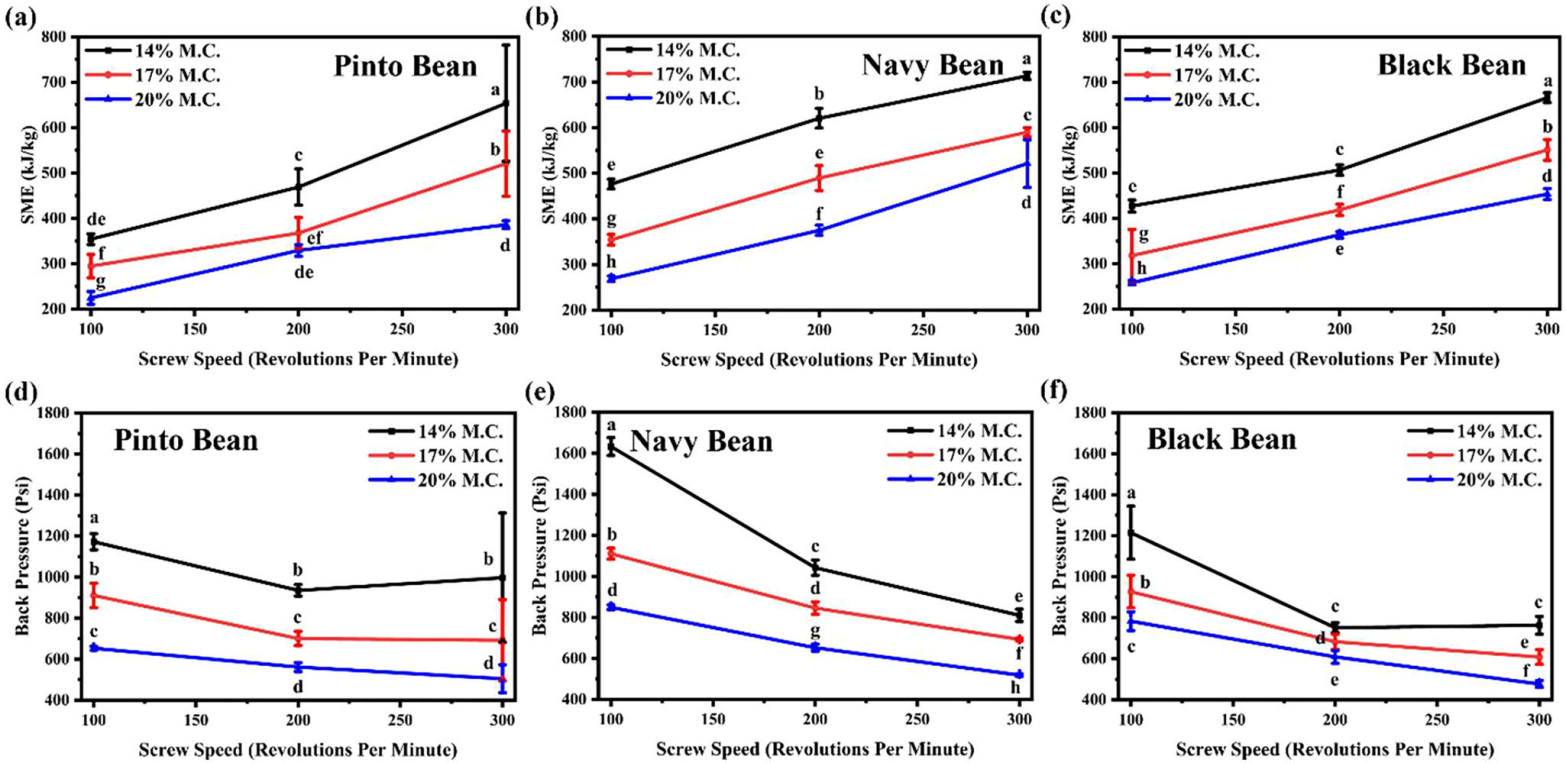
Process responses during extrusion of pinto, navy, and black bean flours at three moisture levels. The measurement results for specific mechanical energy (SME) and back pressure during pinto, navy, and black bean flour extrusion at 14%, 17%, and 20% moisture levels are presented in the figures (black, red, and blue curves, respectively). The graphs in panels (a), (b), and (c) display SME data for pinto and navy and black bean flours, while panels (d), (e), and (f) show back pressure results. The standard deviation values appear as error bars in the data. The Tukey post hoc test at 95% confidence level shows that values with identical letters above error bars in each panel maintain statistical equivalence.

Back pressure (BP), is the maximum pressure response recorded before the die exit at the location shown in Figure 1, which varied inversely with an increase in moisture content and, in general, with an increase in screw speed for all bean flours. From Figure 4d,e,f, it is clear that with a constant screw speed, an increase in moisture content reduced back pressure. Also, at constant moisture, an increase in screw speed reduced back pressure for all the beans. Since the pressure transducer was placed upstream of the die on the die adapter (Figure 1), it can be inferred that the measured values are essentially a measure of the pressure required for forcing materials through the adapter‐die restriction and can thus be taken as an index of resistance to flow due to material melt viscosity (elasticity) for a given restriction. This consistent reduction in back pressure with an increase in water content naturally complements torque behavior and supports a usual explanation, where higher water content results in a consequent decrease in melt viscosity. In addition, an inverse relationship between an increase in screw speed and back pressure can be taken to be normal due to a resultant shear‐thinning effect and resultant increase in temperature in the metering section, thus further lowering upstream pressures in many high‐protein‐high‐fiber mixes. Higher pressures for navy beans were consistent with higher torque values/SME, which can be taken as a measure of greater resistance to flow. Furthermore, high standard deviation levels for pinto bean back pressure at 14% moisture content and 300 rpm indicate process instability at low moisture and high shear rates, where even slight changes in feed rates, slips, and/or partial blockages could accentuate pressurization variation in limited geometric configurations.

### 3.4 Product Physical Properties

The expansion ratio varied by bean type and processing condition, ranging from 1.30 to 2.49 across all tests [Figure 5a,b,c]. The navy bean showed the maximum overall expansion, while the pinto bean had the lowest expansion ratios for all processing conditions. An intermediate pattern was observed in black bean, with a maximum expansion ratio of 2.11 at 14% moisture content and 100 rpm, but significantly decreased with increasing screw speed at 14% moisture content. There was significant interaction among beans under different moisture and screw speed conditions (*p* < 0.05). There was a similar reduction of expansion in all beans due to higher feed moisture content, especially at 100 rpm screw speed. It is well reported in literature that water acts as a plasticizer, which lowers the melt elasticity and viscosity, making the dough less able to trap steam and expand rapidly upon exiting the die (B.‐J. Gu et al. 2020). It reduces the shear forces that create porous structures. The effect of screw speed is dependent on beans and moisture content, where navy and black beans showed lower expansion at 300 rpm values at 14% moisture content, which might be due to reduced residence time and therefore instability in bubble growth under highly viscous conditions at higher shear rates (Fel et al. 2014b; Huang et al. 2022). This phenomenon resulted in decreased expansion due to instability under higher shear rates. In contrast, the pinto bean resulted in a typical increase in expansion due to screw speed because of greater ease of cooking at greater shear rates (B. Gu et al. 2020). Furthermore, Robin et al. (2012) found that the addition of dietary fiber generally resulted in decreased expansion, increased density, and changed texture of extruded products. Generally, insoluble fiber tended to have greater adverse effects than soluble fiber due to incompatibility with the starch matrix and negative effects on bubble growth and collapse. In this study, black bean flour had the highest IDF values, which may have physically disrupted the starch‐protein melt rheology and limited the uniform expansion of bubbles from the die during extrusion. These differences among the bean types were expected to be due to starch type and specific interactions like protein‐starch or fiber‐protein interactions (Simons et al. 2015).

**FIGURE 5:**
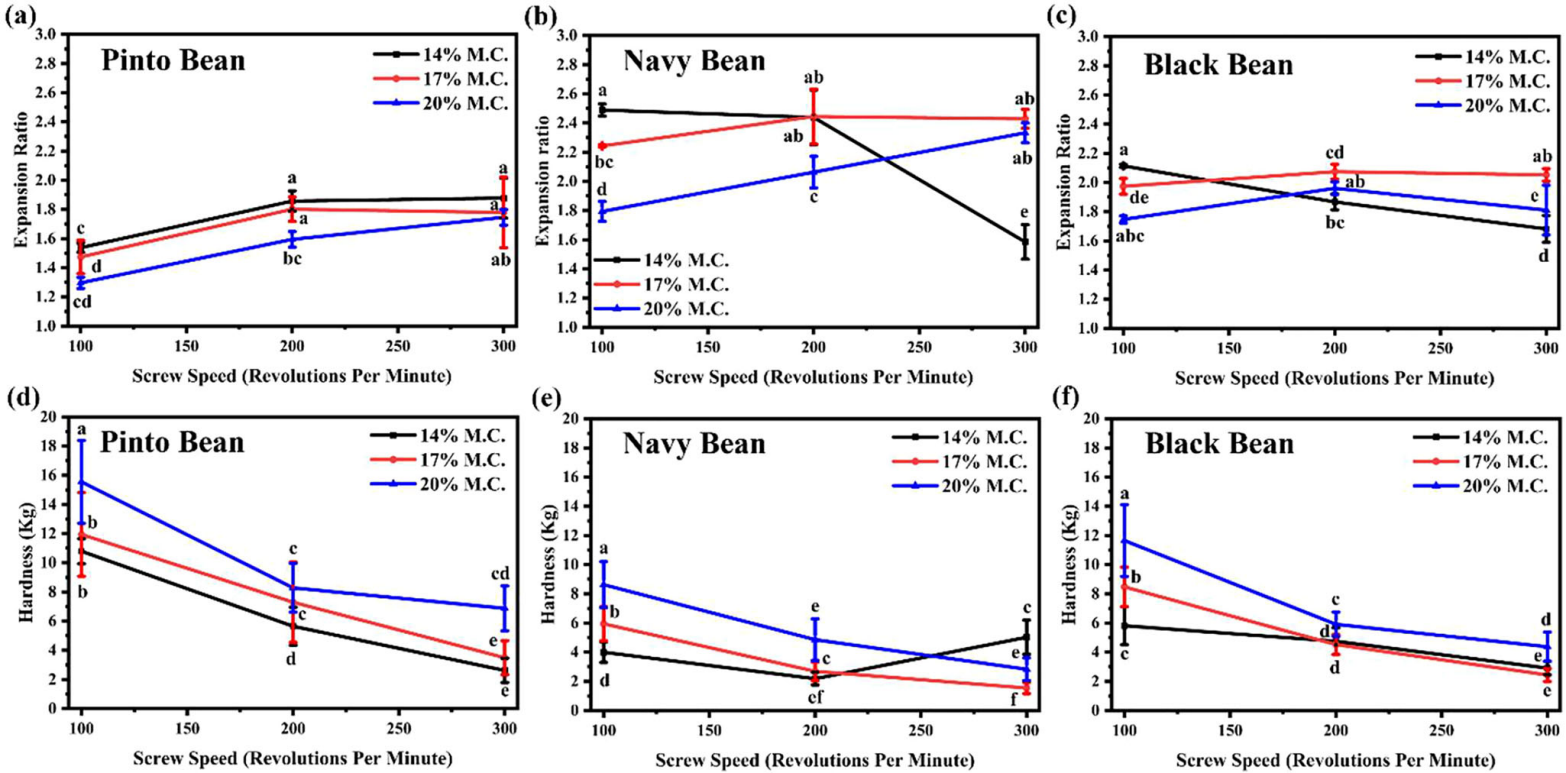
Physical properties of extruded pinto, navy, and black bean products at three moisture levels. The measurement results for expansion ratio and texture hardness of expanded extrudates produced from pinto navy and black bean flour extrusion at 14%, 17%, and 20% moisture levels, respectively (black, red, and blue curves, respectively). The graphs in panels (a), (b), and (c) display expansion ratio measurements for pinto and navy and black bean flours, while panels (d), (e), and (f) show texture hardness data. The standard deviation values appear as error bars in the data. The Tukey post hoc test at 95% confidence level shows that values with identical letters above error bars in each panel maintain statistical equivalence.

The range of hardness was quite large (1.54–15.55 kg) and strongly dependent on the type of bean Figure 5d,e,f. Pinto beans showed the highest hardness among all beans, and the lowest in navy bean extrudates. For all legume types, harder textures generally correlated with higher moisture levels and slower screw speed (8.63 kg, navy, 11.65 kg, black, respectively, both 20% and 100 rpm). There was a similarity between the trends of expansion and texture hardness, as harder textures, especially at high moisture levels and low speed, reflected less expansion. This might be due to higher moisture levels combined with lower speeds, which represented less favorable conditions for expansion, resulting in more compact structures, especially for pinto beans, represented by their generally lowest expansion levels. Any reduction in speed, especially at 17% and 20% moisture, generally corresponded with rising texture hardness. Comparative studies of different fiber fractions and WAI of navy bean and pinto bean flours were also reported in the literature (Moghaddam et al. 2018). Overall, results indicated that navy bean flour contained the lowest fiber fractions, but possessed the highest WAI values, signifying good hydration and the least interference from fiber during melt formation, which further relates it to the expansion stabilization.

### 3.5 Colorimetric Analysis

The color difference between extruded samples and their respective raw flours became smaller with the higher moisture and screw speed (Table 2). Furthermore, the macroscopic visual images are given in the Supplementary File [Figure S1a,b,c]. The ΔE values achieved their highest values with the higher screw speeds in low‐moisture conditions. dry conditions (for example, navy bean 14% moisture content, 300 rpm: 18.53 ± 0.40; pinto bean 14% moisture content, 300 rpm: 13.78 ± 0.86). In contrast, the ΔE values were lowest at high moisture levels and low screw speed (for example, black bean 20% moisture content, 100 rpm: 4.35 ± 1.77; navy bean 20% moisture content, 100 rpm: 8.25 ± 1.36). The color measurements follow a predictable pattern, which shows L^*^ values decreasing while a^*^ and b^*^ values increasing, resulting in higher ΔE values at reduced moisture and elevated screw speed, according to previous studies on extrusion (Gulati et al. 2016; Sun and Muthukumarappan 2002). The assessment of pulse extrusion and high‐protein/high‐fiber extrusion systems showed that these changes happen because of Maillard reactions and other thermal reactions, which become more intense in harsh thermo‐mechanical conditions (Singh et al. 2007). The study supports this mechanism because the conditions that resulted in the biggest ΔE value also led to increased SME in the system, which indicates higher energy input that would speed up browning.

### 3.6 Water Absorption Index (WAI) and Water Solubility Index (WSI)

WAI and WSI exhibited distinct changes in the starch‐protein matrix during extrusion processing. WAI measures the water absorption ability of insoluble materials for gel formation, and WSI measures the amount of soluble compounds that form during processing (Anderson et al. 1970). The WAI range in this study remained small (3.56–4.34) because extrusion processing during the testing period resulted in minimal changes in hydration capacity. The hydration capacity of navy and black beans reached its highest point when feed moisture levels were at their highest because these conditions allowed starch to swell and form a gel, which produced water‐retentive rehydrated matrices (Anderson et al. 1970; Cotacallapa‐Sucapuca et al. 2021). WSI showed a direct relationship with process severity because the WSI values increased when screw speed increased and decreased when moisture levels increased, which indicates that higher shear and thermal forces at low moisture levels break down macromolecules through starch dextrinization and solubilization of small molecules, thus increasing soluble solids (Ek, Kowalski, et al. 2020). It can be concluded that low moisture levels combined with high screw speeds yield the highest SME values and WSI measurements, validating WSI as an effective indicator of extrusion shear intensity in these bean‐based mixtures.

### 3.7 Microstructural Analysis

The extrudates from all treatment conditions showed a foam structure, with air voids separated by a continuous matrix and localized areas of fracture. This is consistent with the concept of bubble nucleation/growth and cell wall stability in extruded melts. At 14% moisture content (Figure 6), more open and coalesced cells (as observed for pinto at 200–300 rpm) typically reflect greater bubble growth and cell merging. Microscopic images of extrudates from 17% and 20% feed moisture content are given in the Supplementary File (Figures S2 and S3). At higher moisture, the melt is expected to be more plasticized, which can promote bubble growth and cell coalescence (larger voids) but can also reduce wall strength and lead to localized collapse or thick, fused lamellae, depending on composition and screw‐speed‐driven shear or energy input. This “more expanded in some regions but more collapsed/lamellar in others” pattern is consistent with how moisture and process severity interact to control cell nucleation, wall thinning, and collapse in pulse‐based expanded extrudates. Overall, the SEM images showed that the structure of expanded bean extrudates is primarily governed by cell formation and stabilization, while processing conditions can alter the extent of porosity and collapse, rather than creating fundamentally different morphologies.

**FIGURE 6:**
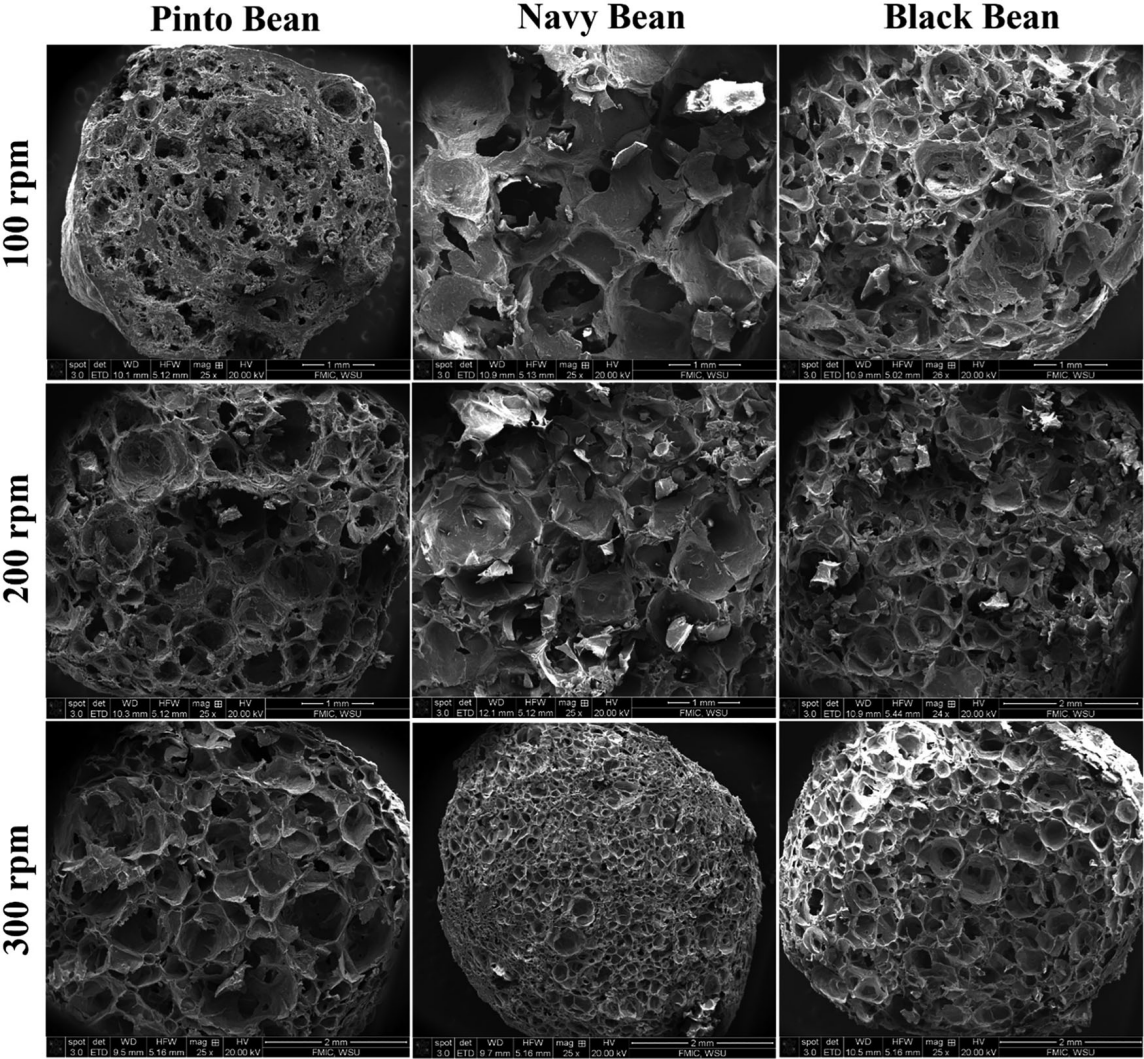
Microscopic SEM images of expanded pinto, navy, and black beans extruded at 14% moisture content with different screw speeds.

### 3.8 Correlation Heat Map

The study showed that WSI was strongly positively correlated with SME across all bean types, while the WAI‐SME relationship was bean dependent. This suggests that WSI can serve as an indicator for measuring extrusion shear intensity and, in turn, macromolecular breakdown or solubilization (Figure 7). The pinto bean expansion‐hardness relationship follows a negative pattern (r = −0.95), which is according to the expected changes in expanded extrudate structure‐texture behavior (Singha et al. 2018). The relationship between SME and expansion depends on the bean type: pinto beans showed a strong correlation (r = 0.83), whereas navy and black beans showed a weak relationship. Therefore, the expansion process depends on bean‐specific matrix properties and their interaction with the processing severity.

**FIGURE 7:**
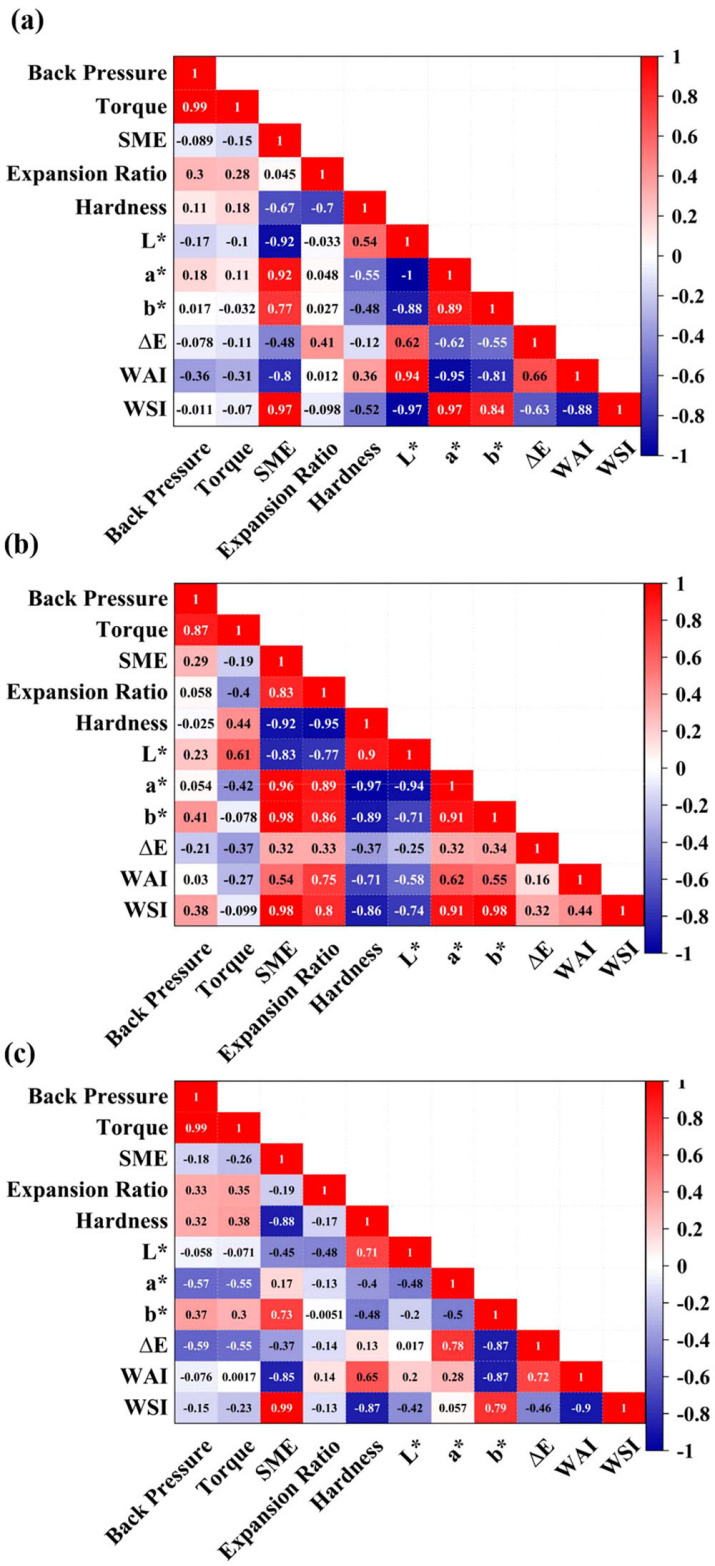
Pearson correlation analysis between extrusion‐processing and product‐quality parameters (back pressure, torque, SME (specific mechanical energy), expansion ratio, hardness, L^*^, a^*^, b^*^, ΔE, WAI, and WSI). Individual heatmaps for: (a) navy bean, (b) pinto bean, and (c) black bean.

### 3.9 K‐Means Clustering

Figure 8 shows K‐means clustering analysis of extrusion processing and product quality parameters (back pressure, torque, SME, expansion ratio, hardness, L^*^, a^*^, b^*^, ΔE, WAI, and WSI). The cluster analysis yielded three groups with 6, 15, and 6 samples in clusters I, II, and III, respectively. The distance measurements among the clusters showed separation among the clusters (Supplementary File: Table S2). The clustering shows the grouping of the sampling sets according to the processing conditions and the product qualities derived from them. K‐means cluster analysis with 11 extrusion‐related variables (back pressure, torque, SME, expansion ratio, hardness, L^*^, a^*^, b^*^, ΔE, WAI, and WSI) was conducted. Based on the elbow point criterion and interpretability, a three‐cluster solution (k = 3) was identified. The cluster mean plot showed distinct standardized response profiles among the three groups. Cluster I was associated with high expansion ratio, L^*^, back pressure, and torque, whereas cluster III was associated with higher SME and WSI. However, cluster II is better described as a lower‐processing‐intensity, low‐expansion, darker‐color group with higher hardness and WAI. The centroid distances indicated separation among the clusters (3.69 for cluster I vs cluster II, 4.27 for cluster I vs cluster III, and 3.59 for cluster II vs cluster III). The sums of squares were 31.6 and 103.3 for clusters III and II, respectively, indicating the highest variability in cluster 2. Cluster I occupied the positive section of PC1 and the lower half of PC2 (gray group). This cluster is described as above average in back pressure, torque, expansion ratio, L^*^, b^*^, ΔE, and WAI, but below average in hardness, SME, a^*^, and WSI, corresponding to more expanded and lighter/yellower extrudates (Table 3). This group primarily contained navy bean blends. The within‐cluster sum of squares value of 38.27 indicated a good homogeneity among these observations. Cluster II strongly grouped around the negative PCI and close to zero PC2 (red group). This cluster tended to have lower mechanical energy inputs, with negative standardized values for back pressure, torque, and SME, and lower expansion and a darker color. This is because these characteristics represent the extrudate products from black bean and pinto bean formulation ingredients, which have higher fiber and phenolic levels that restrict starch expansion and gelation (Wagner and Ganjyal 2024). This cluster showed high variability, with a within cluster sum of squares (WCSS) value of 103.33. The third cluster, located in the upper‐right quadrant of the PCA plot, was associated with higher SME and WSI, indicating a tendency towards extrusion under increased heat and mechanical forces, leading to elevated macromolecular breakdown and solubilization (Hwang et al. 1998). This cluster showed a slight increase in expansion and brighter, yellow colors. This group mainly included navy bean extrudates, with a low WCSS value of 31.59, showing a remarkably consistent reaction under these processing conditions. The distances among the final cluster centroids showed a distinction among the three clusters (Cluster I vs. Cluster II: 3.69 units; Cluster I vs. Cluster III: 4.27 units; and Cluster II vs. Cluster III: 3.59 units), supporting the presence of three distinct multivariate groups including processing conditions and physicochemical characteristics of the extruded products. The clustering indicated an interplay among bean types and severity levels in determining the mechanical energy inputs, physicochemical characteristics, and functionality of the extrudates.

**FIGURE 8:**
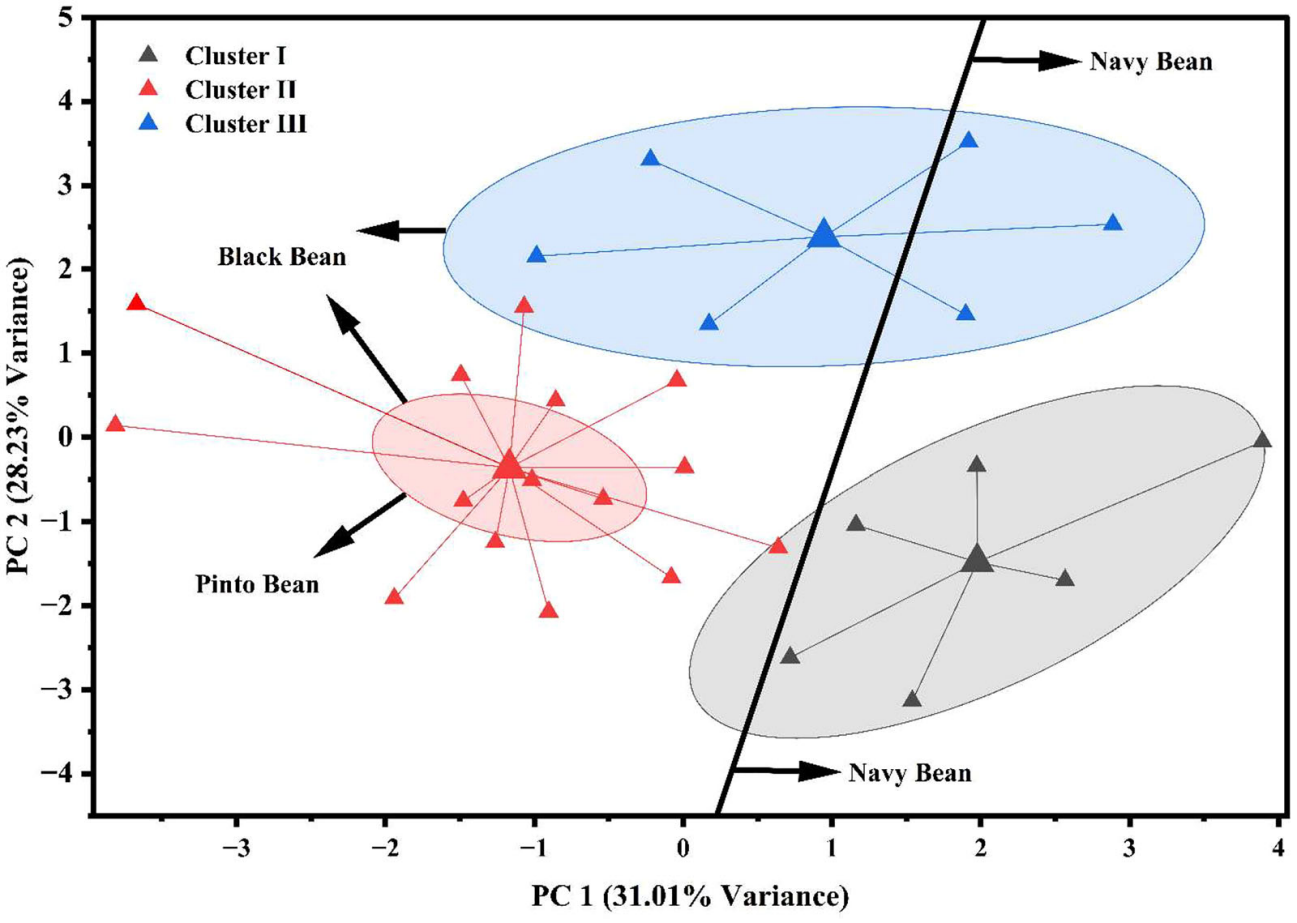
K‐means clustering analysis of extrusion processing and product quality parameters (Back Pressure, Torque, SME, Expansion Ratio, Hardness, L, a*, b*, ΔE, WAI, and WSI). The clustering shows the grouping of the sampling sets according to the processing conditions and the qualities of the products derived from these sets.

**TABLE 3:** Standardized values (z‐scores) of the final cluster centroids obtained from k‐means clustering (k = 3) of extrusion processing and product.

	Back Pressure	Torque	SME	Expansion Ratio	Hardness	L^*^	a^*^	b^*^	∆E	WAI	WSI
Cluster I	0.45017	0.45459	−0.22186	0.93916	−0.32997	1.36233	−0.79512	1.03286	0.70426	0.26260	−0.35524
Cluster II	−0.18090	−0.09673	−0.48517	−0.47897	0.47897	−0.43137	0.15118	−0.67493	−0.05518	0.41589	−0.43700
Cluster III	0.00208	−0.21277	1.43479	0.25827	0.25827	−0.28391	0.41718	0.65448	−0.56631	−1.30231	1.44773

## 4 Conclusion

Black, navy, and pinto bean flours were used to create well‐expanded extrudates. The physical characteristics of these products were controlled by the type of bean and the extrusion processing conditions. The bean extrudates showed unique properties in terms of hardness and expansion. In general, pinto beans generated denser, harder extrudates, while navy beans produced more expanded, softer ones. In contrast, WSI varied considerably more than WAI across all bean varieties, indicating that solubility is more susceptible to extrusion severity than water absorption. The DSC findings, however, demonstrated that all of the raw bean mixes had similar thermal transition ranges. However, the microscopic structure and corresponding statistical analysis of the samples revealed that the type of bean used in the product was crucial in determining the development of the cellular structure, the color change, and the texture of the final product. Consequently, optimal bean‐specific extrusion conditions need to be determined to develop high‐protein, high‐fiber, well‐expanded products. Among the three bean flours, navy bean flour showed the best overall performance for expanded product development because it produced the highest expansion and the lowest hardness under the tested conditions. The general trend of lower feed moisture and higher screw speed improved the expansion, and product structure was observed within the range of the processing conditions studied. The outcome of this study can be utilized for the selection of bean flour as a commercial pulse ingredient for the development of ready‐to‐eat expanded foods. While the composition of bean flours can serve as a criterion for selecting raw materials for developing functional pulse‐based extruded foods, additional characteristics, such as hydration behavior, dietary fiber distribution, and thermal behavior, should also be assessed.

## Author Contributions

**Aniket Kamboj**: conceptualization, methodology, investigation, data curation, validation, visualization, writing – original draft. **Aimé Roger Raherison**: data curation, writing – review and editing. **Sahil Nain**: data curation, writing – review and editing. **Girish M. Ganjyal**: conceptualization, funding acquisition, resources, supervision, writing – review and editing.

## Conflicts of Interest

The authors declare no conflicts of interest.

## Supporting information




**Supplementary Material**: jfds71537‐sup‐0001‐SuppMat.docx

## References

[jfds71537-bib-0001] Anderson, R. A. , H. F. Conway , and A. J. Peplinski . 1970. “Gelatinization of Corn Grits by Roll Cooking, Extrusion Cooking and Steaming.” Starch—Stärke 22, no. 4: 130–135. 10.1002/star.19700220408.

[jfds71537-bib-0002] Bernin, J. , P. Watanabe , C. E. Wagner , S. Smith , and G. M. Ganjyal . 2024. “Mung Bean Protein Enhances the Expansion of Corn Starch During Twin‐Screw Extrusion.” Journal of Food Science 89, no. 12: 9379–9391. 10.1111/1750-3841.17375. PMC11673559 39495597

[jfds71537-bib-0003] Boakye, P. G. , A. Y. Okyere , I. Kougblenou , R. Kowalski , B. P. Ismail , and G. A. Annor . 2022. “Optimizing the Extrusion Conditions for the Production of Expanded Intermediate Wheatgrass (*Thinopyrum intermedium*) Products.” Journal of Food Science 87, no. 8: 3496–3512. 10.1111/1750-3841.16238. PMC9541489 35781707

[jfds71537-bib-0004] Chung, H.‐J. , Q. Liu , R. Hoover , T. D. Warkentin , and B. Vandenberg . 2008. “In Vitro Starch Digestibility, Expected Glycemic Index, and Thermal and Pasting Properties of Flours From Pea, Lentil and Chickpea Cultivars.” Food Chemistry 111, no. 2: 316–321. 10.1016/j.foodchem.2008.03.062. 26047429

[jfds71537-bib-0005] Cotacallapa‐Sucapuca, M. , E. N. Vega , H. A. Maieves , et al. 2021. “Extrusion Process as an Alternative to Improve Pulses Products Consumption. A Review.” Foods 10, no. 5: 1096. 10.3390/foods10051096. PMC8156340 34063375

[jfds71537-bib-0006] Ding, Q.‐B. , P. Ainsworth , G. Tucker , and H. Marson . 2005. “The Effect of Extrusion Conditions on the Physicochemical Properties and Sensory Characteristics of Rice‐Based Expanded Snacks.” Journal of Food Engineering 66, no. 3: 283–289. 10.1016/j.jfoodeng.2004.03.019.

[jfds71537-bib-0007] Dogan, H. , A. Gueven , and Z. Hicsasmaz . 2013. “Extrusion Cooking of Lentil Flour (*Lens culinaris* –Red)–Corn Starch–Corn Oil Mixtures.” International Journal of Food Properties 16, no. 2: 341–358. 10.1080/10942912.2011.551866.

[jfds71537-bib-0008] Du, S. , H. Jiang , X. Yu , and J. Jane . 2014. “Physicochemical and Functional Properties of Whole Legume Flour.” LWT—Food Science and Technology 55, no. 1: 308–313. 10.1016/j.lwt.2013.06.001.

[jfds71537-bib-0009] Ek, P. , J. M. Baner , and G. M. Ganjyal . 2020. “Extrusion Processing of Cereal Grains, Tubers, and Seeds.” In Extrusion Cooking, 225–263. Elsevier. 10.1016/B978-0-12-815360-4.00008-0.

[jfds71537-bib-0010] Ek, P. , B. Gu , and G. M. Ganjyal . 2021. “Whole Seed Lentil Flours From Different Varieties (Brewer, Crimson, and Richlea) Demonstrated Significant Variations in Their Expansion Characteristics During Extrusion.” Journal of Food Science 86, no. 3: 942–951. 10.1111/1750-3841.15623. 33565641

[jfds71537-bib-0011] Ek, P. , R. J. Kowalski , and G. M. Ganjyal . 2020. “Raw Material Behaviors in Extrusion Processing I (Carbohydrates).” In Extrusion Cooking, 119–152. Elsevier. 10.1016/B978-0-12-815360-4.00004-3.

[jfds71537-bib-0012] Fel, E. , V. Massardier , F. Mélis , B. Vergnes , and P. Cassagnau . 2014a. “Residence Time Distribution in a High Shear Twin Screw Extruder.” International Polymer Processing 29, no. 1: 71–80. 10.3139/217.2805.

[jfds71537-bib-0013] Fel, E. , V. Massardier , F. Mélis , B. Vergnes , and P. Cassagnau . 2014b. “Residence Time Distribution in a High Shear Twin Screw Extruder.” International Polymer Processing 29, no. 1: 71–80. 10.3139/217.2805.

[jfds71537-bib-0014] Ferawati, F. , M. Hefni , and C. Witthöft . 2019. “Flours From Swedish Pulses: Effects of Treatment on Functional Properties and Nutrient Content.” Food Science & Nutrition 7, no. 12: 4116–4126. 10.1002/fsn3.1280. PMC6924304 31890191

[jfds71537-bib-0015] Ganjyal, G. M. 2020. “Overview.” In Extrusion Cooking (xix–xxi), edited by G. M. Ganjyal. Woodhead Publishing. 10.1016/B978-0-12-815360-4.10000-8.

[jfds71537-bib-0016] Ganjyal, G. M. , M. A. Hanna , and D. D. Jones . 2003. “Modeling Selected Properties of Extruded Waxy Maize Cross‐Linked Starches With Neural Networks.” Journal of Food Science 68, no. 4: 1384–1388. 10.1111/j.1365-2621.2003.tb09654.x.

[jfds71537-bib-0017] Gu, B. , M. D. P. Masli , and G. M. Ganjyal . 2020. “Whole Faba Bean Flour Exhibits Unique Expansion Characteristics Relative to the Whole Flours of Lima, Pinto, and Red Kidney Beans During Extrusion.” Journal of Food Science 85, no. 2: 404–413. 10.1111/1750-3841.14951. 31887250

[jfds71537-bib-0018] Gu, B.‐J. , M. P. Wolcott , and G. M. Ganjyal . 2020. “Optimized Screw Profile Design Proved to Inhibit Re‐Agglomeration That Occurs During Extrusion of Fine‐Milled Forest Residuals for Producing Fermentable Sugars.” Industrial Crops and Products 154: 112730. 10.1016/j.indcrop.2020.112730.

[jfds71537-bib-0019] Gulati, P. , S. A. Weier , D. Santra , J. Subbiah , and D. J. Rose . 2016. “Effects of Feed Moisture and Extruder Screw Speed and Temperature on Physical Characteristics and Antioxidant Activity of Extruded Proso Millet (*Panicum miliaceum*) Flour.” International Journal of Food Science & Technology 51, no. 1: 114–122. 10.1111/ijfs.12974.

[jfds71537-bib-0020] Hoover, R. , T. Hughes , H. J. Chung , and Q. Liu . 2010. “Composition, Molecular Structure, Properties, and Modification of Pulse Starches: A Review.” Food Research International 43, no. 2: 399–413. 10.1016/j.foodres.2009.09.001.

[jfds71537-bib-0021] Hoover, R. , and W. S. Ratnayake . 2002. “Starch Characteristics of Black Bean, Chick Pea, Lentil, Navy Bean and Pinto Bean Cultivars Grown in Canada.” Food Chemistry 78, no. 4: 489–498. 10.1016/S0308-8146(02)00163-2.

[jfds71537-bib-0022] Huang, Z. , D. Wu , L. Xu , Y. Yang , and Y. Shuai . 2022. “Effects of Shear Stress on the Behavior of Volatile Bubbles in the High‐Density Polyethylene Melt.” Polymer Engineering & Science 62, no. 11: 3759–3772. 10.1002/pen.26142.

[jfds71537-bib-0023] Hwang, J.‐K. , J.‐S. Choi , C.‐J. Kim , and C.‐T. Kim . 1998. “Solubilization of Apple Pomace by Extrusion.” Journal of Food Processing and Preservation 22, no. 6: 477–491. 10.1111/j.1745-4549.1998.tb00364.x.

[jfds71537-bib-0024] Jeganathan, B. , and G. M. Ganjyal . 2026. “Plant‐Based Proteins in Snacks: Challenges and Opportunities.” In Plant Proteins, (237–260). Elsevier. 10.1016/B978-0-323-91725-4.00017-5.

[jfds71537-bib-0025] Kamboj, A. , J. K. Richter , C. Lee , et al. 2025. “Azodicarbonamide, Hydrogen Peroxide, and L ‐Ascorbic Acid Aid in the Modification of Protein Texture during High‐Moisture Meat Analog Extrusion Processing.” Journal of Food Science 90, no. 7: e70346. 10.1111/1750-3841.70346. PMC12226206 40610193

[jfds71537-bib-0026] Kowalski, R. J. , J. P. Hause , H. Joyner (Melito) , and G. M. Ganjyal . 2018. “Waxy Flour Degradation—Impact of Screw Geometry and Specific Mechanical Energy in a Co‐Rotating Twin Screw Extruder.” Food Chemistry 239: 688–696. 10.1016/j.foodchem.2017.06.120. 28873623

[jfds71537-bib-0027] Kowalski, R. J. , C. F. Morris , and G. M. Ganjyal . 2015. “Waxy Soft White Wheat: Extrusion Characteristics and Thermal and Rheological Properties.” Cereal Chemistry 92, no. 2: 145–153. 10.1094/CCHEM-03-14-0039-R.

[jfds71537-bib-0028] Kyomugasho, C. , P. G. Kamau , S. Aravindakshan , and M. E. Hendrickx . 2021. “Evaluation of Storage Stability of Low Moisture Whole Common Beans and Their Fractions Through the Use of State Diagrams.” Food Research International 140: 109794. 10.1016/j.foodres.2020.109794. 33648161

[jfds71537-bib-0029] Lee Rodgers, J. , and W. A. Nicewander . 1988. “Thirteen Ways to Look at the Correlation Coefficient.” The American Statistician 42, no. 1: 59–66. 10.1080/00031305.1988.10475524.

[jfds71537-bib-0030] Lefèvre, C. , P. Bohuon , L. Akissoé , L. Ollier , B. Matignon , and C. Mestres . 2021. “Modeling the Gelatinization‐Melting Transition of the Starch‐Water System in Pulses (Lentil, Bean and Chickpea).” Carbohydrate Polymers 264: 117983. 10.1016/j.carbpol.2021.117983. 33910712

[jfds71537-bib-0031] Lefèvre, C. , P. Bohuon , V. Lullien‐Pellerin , and C. Mestres . 2022. “Modeling the Thermal Denaturation of the Protein–Water System in Pulses (Lentils, Beans, and Chickpeas).” Journal of Agricultural and Food Chemistry 70, no. 32: 9980–9989. 10.1021/acs.jafc.2c03553. 35921686

[jfds71537-bib-0032] Li, C. , R. J. Kowalski , L. Li , and G. M. Ganjyal . 2017. “Extrusion Expansion Characteristics of Samples of Select Varieties of Whole Yellow and Green Dry Pea Flours.” Cereal Chemistry 94, no. 3: 385–391. 10.1094/CCHEM-04-16-0079-R.

[jfds71537-bib-0033] Lloyd, S. 1982. “Least Squares Quantization in PCM.” IEEE Transactions on Information Theory 28, no. 2: 129–137.

[jfds71537-bib-0034] McQueen, J. B. 1967. “Some Methods of Classification and Analysis of Multivariate Observations.” In Proceedings of the Fifth Berkeley Symposium on Mathematical Statistics and Probability , 281–297. University of California Press. https://cir.nii.ac.jp/crid/1571135649659368064.

[jfds71537-bib-0035] Moghaddam, S. M. , M. A. Brick , D. Echeverria , et al. 2018. “Genetic Architecture of Dietary Fiber and Oligosaccharide Content in a Middle American Panel of Edible Dry Bean.” The Plant Genome 11, no. 1: 170074. 10.3835/plantgenome2017.08.0074. PMC12962584 29505640

[jfds71537-bib-0036] Patrascu, L. , I. Vasilean , I. Banu , and I. Aprodu . 2017. “Functional Properties of Pulse Flours and Their Opportunities in Spreadable Food Products.” Quality Assurance and Safety of Crops & Foods 9, no. 1: 67–78.

[jfds71537-bib-0037] Pietrysiak, E. , Y. Zhu , B. Gu , and G. M. Ganjyal . 2020. “Whole Nuña Bean (*Phaseolus vulgaris* L.) Flour Showed Higher Direct Expansion During Extrusion Processing at Relatively Lower Temperatures.” Journal of Food Science 85, no. 7: 2134–2142. 10.1111/1750-3841.15180. 32506502

[jfds71537-bib-0038] Ratnayake, W. S. , and D. S. Jackson . 2008. “Starch Gelatinization.” In Advances in Food and Nutrition Research, 221–268. Elsevier. 10.1016/S1043-4526(08)00405-1. 18772106

[jfds71537-bib-0039] Robin, F. , H. P. Schuchmann , and S. Palzer . 2012. “Dietary Fiber in Extruded Cereals: Limitations and Opportunities.” Trends in Food Science & Technology 28, no. 1: 23–32. 10.1016/j.tifs.2012.06.008.

[jfds71537-bib-0040] Simons, C. W. , C. Hall , M. Tulbek , M. Mendis , T. Heck , and S. Ogunyemi . 2015. “Acceptability and Characterization of Extruded Pinto, Navy and Black Beans.” Journal of the Science of Food and Agriculture 95, no. 11: 2287–2291. 10.1002/jsfa.6948. 25298221

[jfds71537-bib-0041] Singh, S. , S. Gamlath , and L. Wakeling . 2007. “Nutritional Aspects of Food Extrusion: A Review.” International Journal of Food Science & Technology 42, no. 8: 916–929. 10.1111/j.1365-2621.2006.01309.x.

[jfds71537-bib-0042] Singha, P. , K. Muthukumarappan , and P. Krishnan . 2018. “Influence of Processing Conditions on Apparent Viscosity and System Parameters During Extrusion of Distiller's Dried Grains‐Based Snacks.” Food Science & Nutrition 6, no. 1: 101–110. 10.1002/fsn3.534. PMC5778219 29387367

[jfds71537-bib-0043] Siva, N. , P. Thavarajah , S. Kumar , and D. Thavarajah . 2019. “Variability in Prebiotic Carbohydrates in Different Market Classes of Chickpea, Common Bean, and Lentil Collected from the American Local Market.” Frontiers in Nutrition 6: 38. 10.3389/fnut.2019.00038. PMC6462997 31024922

[jfds71537-bib-0044] Slade, L. , and H. Levine . 1995. “Glass Transitions and Water‐Food Structure Interactions.” In Advances in Food and Nutrition Research, 103–269. Elsevier. 10.1016/S1043-4526(08)60084-4. 15918292

[jfds71537-bib-0045] Stone, A. K. , M. G. Nosworthy , C. Chiremba , J. D. House , and M. T. Nickerson . 2019. “A Comparative Study of the Functionality and Protein Quality of a Variety of Legume and Cereal Flours.” Cereal Chemistry 96, no. 6: 1159–1169. 10.1002/cche.10226.

[jfds71537-bib-0046] Stone, A. K. , S. Parolia , J. D. House , N. Wang , and M. T. Nickerson . 2021. “Effect of Roasting Pulse Seeds at Different Tempering Moisture on the Flour Functional Properties and Nutritional Quality.” Food Research International 147: 110489. 10.1016/j.foodres.2021.110489 34399485

[jfds71537-bib-0047] Sun, Y. , and K. Muthukumarappan . 2002. “Changes in Functionality of Soy‐Based Extrudates During Single‐Screw Extrusion Processing.” International Journal of Food Properties 5, no. 2: 379–389. 10.1081/JFP-120005793.

[jfds71537-bib-0048] Thakur, S. , M. G. Scanlon , R. T. Tyler , A. Milani , and J. Paliwal . 2019. “Pulse Flour Characteristics From a Wheat Flour Miller's Perspective: A Comprehensive Review.” Comprehensive Reviews in Food Science and Food Safety 18, no. 3: 775–797. 10.1111/1541-4337.12413. 33336925

[jfds71537-bib-0049] Wagner, C. E. , and G. M. Ganjyal . 2024. “Impact of Functional Dietary Fiber Incorporation on the Appearance and Mechanical Properties of Extruded High Moisture Meat Analogs.” Journal of Food Science 89, no. 8: 4953–4968. 10.1111/1750-3841.17164. 38980955

[jfds71537-bib-0050] Wagner, C. E. , J. K. Richter , D. Dey , S. Finnie , and G. M. Ganjyal . 2023. “Impact of Tamarind Seed Gum on the Viscosity Behavior, Thermal Properties, and Extrusion Characteristics of Native Corn Starch.” Journal of Food Science 88, no. 4: 1595–1609. 10.1111/1750-3841.16513. 36883972

[jfds71537-bib-0051] Wagner, C. E. , J. K. Richter , M. Ikuse , and G. M. Ganjyal . 2024. “Classification of Select Functional Dietary Fiber Ingredients Based on Quantitative Properties and Latent Qualitative Criteria.” Journal of Food Science 89, no. 10: 6098–6112. 10.1111/1750-3841.17238. 39206748

[jfds71537-bib-0052] Yeh, A. , and Y. Jaw . 1999. “Effects of Feed Rate and Screw Speed on Operating Characteristics and Extrudate Properties During Single‐Screw Extrusion Cooking of Rice Flour.” Cereal Chemistry 76, no. 2: 236–242. 10.1094/CCHEM.1999.76.2.236.

