# Supplementary material for: Bean Type Strongly Drives Specific Mechanical Energy and Distinguishes Extrudate Expansion During Extrusion Processing
Source: J Food Sci. 2026 Oct 9;91(10):e71537. doi: 10.1111/1750-3841.71537 (PMC13651920; doi:10.1111/1750-3841.71537)
Supplement: Supplementary file 1 [file JFDS-91-0-s001.docx]

**Supplementary information: Bean type strongly drives specific mechanical energy and distinguishes extrudate properties in the direct expansion process during extrusion**

Aniket Kamboj^a^, Aimé Roger Raherison^b^, Sahil Nain^a^, Girish M Ganjyal^a*^

^a^School of Food Science, Washington State University, Pullman, WA, USA

^b^School of Bioresources and Technology, King Mongkut’s University of Technology Thonburi, Bangkok, Thailand

**TABLE S1**: K-means cluster summary (n, within-cluster sum of squares, mean, and maximum distance).

|  | Number of Observations | Within Cluster Sum of Squares | Average Distance | Maximum Distance |
| --- | --- | --- | --- | --- |
| Cluster I | 6 | 38.27 | 2.39 | 4.06 |
| Cluster II | 15 | 103.32 | 2.51 | 4.07 |
| Cluster III | 6 | 31.59 | 2.29 | 2.55 |

**TABLE S2**: Distance between final cluster centers.

|  | Cluster I | Cluster II | Cluster III |
| --- | --- | --- | --- |
| Cluster I | 0.00 | 3.69 | 4.26 |
| Cluster II | 3.69 | 0.00 | 3.59 |
| Cluster III | 4.26 | 3.59 | 0.00 |

**FIGURE S1.** Visual macroscopic appearance of extrudates produced from pinto, navy, and black bean flours at 14% (a), 17% (b), and 20% (c) feed moisture content under varying screw speeds. Scale bars indicate centimeters (cm), with major tick marks representing 1 cm intervals.

**FIGURE S2.** Microscopic SEM images of expanded pinto, navy, and black beans extruded at 17% moisture content with different screw speeds.

**FIGURE S3.** Microscopic SEM images of expanded pinto, navy, and black beans extruded at 20% moisture content with different screw speeds.
